# Sexual Minority Orientation Is Associated With Greater Psychological Impact Due to the COVID-19 Crisis—Evidence From a Longitudinal Cohort Study of Young Swiss Men

**DOI:** 10.3389/fpubh.2021.692884

**Published:** 2021-10-22

**Authors:** Simon Marmet, Matthias Wicki, Gerhard Gmel, Céline Gachoud, Nicolas Bertholet, Joseph Studer

**Affiliations:** ^1^Addiction Medicine, Lausanne University Hospital and University of Lausanne, Lausanne, Switzerland; ^2^Addiction Switzerland, Lausanne, Switzerland; ^3^Centre for Addiction and Mental Health, Toronto, ON, Canada; ^4^Faculty of Health and Social Sciences, University of the West of England, Bristol, United Kingdom

**Keywords:** COVID-19, Switzerland, mental health, sexual orientation, sexual minorities

## Abstract

**Background:** The COVID-19 pandemic and its countermeasures may have had a significant impact on the psychological well-being of specific population subgroups. The present study investigated whether sexual minority men (defined here as attracted partly or exclusively to men) from an ongoing cohort study of young Swiss men experienced different psychological impacts, levels of substance use and addictive behaviors, and to which degree pre-existing vulnerabilities and participants experiences during the crisis might explain these differences.

**Methods:** An ongoing cohort sample based on the general population of young Swiss men (mean age = 29.07 years; SD = 1.27) was assessed before and during the COVID-19 crisis for depression, stress, sleep quality, substance use and addictive behaviors. Additionally, during the crisis, we assessed its impact in form of fear, isolation and traumatic experiences. Potential associations between these outcomes and sexual orientation (sexual minority vs. heterosexual) were tested using linear regression models. It was additionally estimated to which degree these associations were attenuated if adjusted for differences in mental health, personality and socioeconomic status before the crisis, as well as the experience of the COVID-19 crisis (infection with the virus and changes to work situation).

**Results:** Compared to heterosexual men, sexual minority men showed higher levels of psychological trauma (*b* = 0.37 [0.25, 0.49]), fear (*b* = 0.18 [0.06, 0.30]) and isolation (*b* = 0.32 [0.20, 0.44]) due to the COVID-19 pandemic as well as higher levels of depression (*b* = 0.31 [0.20, 0.41]) and lower sleep quality (*b* = −0.13 [−0.24, −0.02]) during the crisis. These differences were to a large degree explained by higher pre-crisis levels of mental health problems and the personality dimension of neuroticism-anxiety. Sexual minority men showed higher overall levels of substance use and addictive behaviors, but these differences were already present before the crisis.

**Conclusion:** The COVID-19 crisis may have worsened pre-existing vulnerabilities in sexual minority men, leading to its greater psychological impact on them than on heterosexual men. Reducing minority stress due to sexual orientation may help not only to improve mental health among important proportions of the population but also to reduce their vulnerability to crises. Services offering psychological support to sexual minorities may need to be reinforced during crises.

## Introduction

The coronavirus disease 2019 (COVID-19) pandemic caused by the severe acute respiratory syndrome coronavirus 2 (SARS-CoV-2) affected daily life worldwide, including in Switzerland. Although infection and potential infection with the virus had evident impacts on the population's physical health, other consequences could be observed in the form of the psychological stress due to fears for one's health and that of others, the measures taken against the spread of the coronavirus, and even the financial strains of economic uncertainty. Cases of COVID-19 started to increase in Switzerland at the beginning of March 2020, with 3,747 confirmed cases (43.9 cases per 100,000 inhabitants) reported by 16 March (1). The Swiss government took drastic measures to halt the spread of infection (hereafter: *COVID measures*), including closing schools, restaurants, non-essential shops, tourist attractions and others sites and introducing social/physical distancing (groups of up to 5 people and at least 2 m apart). There were also strong recommendations to work from home and avoid public transport.

Health crises often highlight and amplify pre-existing vulnerabilities (2–4), potentially also in individuals with a non-heterosexual sexual orientation, henceforth called sexual minorities here. The American Psychological Association defines sexual orientation as “…an enduring pattern of emotional, romantic and/or sexual attractions to men, women or both sexes. Sexual orientation also refers to a person's sense of identity based on those attractions, related behaviors and membership in a community of others who share those attractions.” (5) For the purpose of this study, sexual minority is defined as men which are partly or exclusively attracted to men. Being a member of a sexual minority is associated with minority stress (6–10), which Meyer (10) defines as the excess stress social minorities (in this case due to their sexual minority orientation) suffer due to effects such as stigmatization and prejudice. Stress due to sexual minority status can be categorized in four dimensions (10): external/objective stressors (such as stigma, prejudice or violence), expectation of such external stressors, internalization of negative societal attitudes and concealment of sexual orientation. Minority stress has been related to worse mental health (6, 10–15). Studies have also reported associations between personality traits and sexual orientation (16–18), and high scores for some personality traits, notably neuroticism, which have also been correlated with mental health problems (18). Despite the increased prevalence of mental health problems in sexual minorities, compared to heterosexual individuals, it is important to acknowledge that the majority of sexual minority individuals do not suffer from a mental health problem (13).

Consistent with this literature, previous research (14) on our cohort's sample also found that, compared to heterosexual men, sexual minority men reported worse mental health and overall higher levels of substance use disorders and behavioral addictions. Such pre-existing differences in mental health may predispose sexual minorities to worse reactions to a crisis such as COVID-19 (6, 19). Additionally, sexual minority status is often associated with differences in sociodemographic factors that may also contribute to a worse reaction to crises. For example, sexual minorities may have different housing situations, i.e., living less often with children and more often alone. Not having to care for children during such a crisis may be less stressful for some, but may also make them more prone to social isolation. Sexual minorities often have a lower socioeconomic status (20, 21) than heterosexual people, which could also relate to greater psychological impacts in a crisis (22).

Group solidarity and cohesiveness may provide resilience against negative consequences of minority stress (10), however, these resources may be less present due to the breakdown of usual social structures during the COVID-19 crisis. Conversely, the reduced number of social contacts during the COVID-19 crisis may reduce the occurrence of discriminatory experiences due to sexual orientation.

Overall, these factors may predispose sexual minorities to a worse reaction to the crisis (6, 8) and their existing higher addictive behaviors and substance use (14) may also be further exacerbated as a coping mechanism for COVID-19's psychological impact. Recent research has indicated that members of sexual minority groups have suffered a high psychological impact from the crisis (23–31), but these studies were done in samples of sexual minority people only and, therefore, that impact cannot be compared with the psychological impact suffered by the heterosexual people also affected by the crisis (32). Seven studies with online recruited convenience samples from the United States of America (9, 33–38) and one online study conducted in Portugal and Brazil (39) comparing sexual minorities and heterosexual persons found that the psychological impacts among members of sexual minorities were greater. A prospective cohort study in Southern California also found that sexual minorities reported more negative coping strategies with respect to the COVID-19 crisis compared to heterosexual individuals (40). A review of the literature regarding well-being of sexual and gender minorities in the United Kingdoms identified no research published in scientific journals, but several gray literature reports that overall showed poor or worse outcomes in sexual minorities (41).

Earlier publications have reported on the overall psychological impact of the COVID-19 crisis on our sample of young men, including changes in addictive behaviors and substance use (22, 42, 43). Overall, few participants tested positive for COVID-19, but there were considerable changes in work situations (job loss, partial unemployment, and working from home) due to the crisis and substantial psychological impacts (22, 42). Although there was a decrease in alcohol use, there was a marked increase in non-substance related addictive behaviors, such as gaming and watching TV series (43).

The present study focused on associations between sexual orientation and these outcomes. Specifically, the study's primary aim was to investigate whether sexual orientation could be associated with COVID-19's impacts (on mental well-being, substance use and addictive behaviors). A secondary aim was to better understand why sexual orientation was associated with these outcome variables and which parts of these associations could be accounted for by other covariates measured in our study. Therefore, as a first step, we tested whether sexual orientation was associated with: (a) experiences related to COVID-19; (b) sociodemographic factors; (c) indicators of mental health before the crisis; and (d) personality traits before the crisis. In a second step we also tested the degree to which these covariates accounted for the associations between sexual orientation and psychological impacts, substance use and addictive behaviors.

## Method

### Sample

This study was based on data from the Cohort Study on Substance-Use Risk Factors (C-SURF), collected from waves shortly before and during the COVID crisis. Participants were first contacted when they were about 19 years old, during the mandatory recruitment procedures testing their aptitude for military service. They were recruited at three of the six national military recruitment centers (in Lausanne, Windisch and Mels), which together cover 21 of Switzerland's 26 cantons. Subsequent data collection was independent of the army. The Human Research Ethics Committee of the Canton of Vaud approved the research protocols for the parent C-SURF study and the present COVID study (protocol 15/07 (PB_2018-00296). In total, 13,237 participants were invited to participate in the study, of which 7,556 participants gave their written informed consent and 5,987 replied to the first wave (see https://www.c-surf.ch/en/1.html and (44, 45) for more details about the parent study's design). There have been four waves of assessment to date (at roughly the ages of 20, 21, 25, and 28) with excellent follow up participation rates of about 90%. Between April 2019 and 14 February 2020 (1 month before the announcement of the COVID-19 measures, data collection of the 4th wave was still ongoing) 4,407 participants replied to C-SURF's fourth-wave, online questionnaire (hereafter: the *pre-COVID wave*). On 13 May 2020, these 4,407 participants were sent an e-mail and a short text message by telephone, inviting them to answer the COVID wave assessment. Data was collected online using LimeSurvey software (46), and collection ended on 8 June 2020. A total of 2,548 (57.8%) participants provided their informed consent to participate in the COVID assessment, and 2,415 (54.8% of those invited) filled out at least the first section about their experiences of COVID-19 symptoms and their personal situation. Participants with missing values for predictor variables were excluded from further analysis, and the study's final sample size was 2,345 (53.2% of those invited) participants.

## Measurements

### Sexual Orientation

One question on sexual orientation in the pre-COVID wave asked about the extent to which participants were attracted to men and women, with five response options ranging from exclusively attracted to women to exclusively attracted to men (47, 48). This was recoded to heterosexual (exclusively attracted to women) vs. sexual minority (at least somewhat attracted to men to exclusively attracted to men). Subgroup sample sizes were too small to meaningfully investigate differences across the spectrum of sexual minority orientations. Nevertheless, results for the five-option spectrum of sexual orientations are presented in the Supplementary Materials.

### The Impact of the COVID-19 Pandemic (Outcome Variables)

#### Psychological Consequences, With No Mention of COVID-19 as Their Cause (Measured Before and During COVID-19)

These variables were investigated in the same format in both the pre-COVID and COVID-19 assessments, with COVID-19 not explicitly mentioned as a potential cause. Symptoms of major depression in the past 2 weeks were measured using the 12-item Major Depression Inventory (WHO–MDI) (49, 50), which was recoded into 10 criteria, resulting in a sum score ranging from 0 to 50. Cronbach's Alpha for the depression scale was 0.906 at the pre-COVID wave and 0.914 during COVID-19. Perceived stress in the last month was measured using the four-item short version of the Perceived Stress Scale (51), with response options from 0 (“never”) to 6 (“very often”), resulting in a sum score ranging from 0 to 24. Cronbach's Alpha for this scale was 0.659 at the pre-COVID and 0.656 during COVID. Sleep quality in the last month was assessed with one question from the Pittsburgh Sleep Quality Index (52), and response options from 0 (“very bad”) to 3 (“very good”).

#### Psychological Consequences, Explicitly Mentioning COVID-19 as Their Cause (Only Measured During COVID-19)

These variables explicitly mentioned the COVID-19 pandemic and its countermeasures as their cause, using formulations such as “due to the COVID-19 crisis, I experienced….” Psychological trauma due to the COVID-19 crisis in the last seven days was measured using the 22-item Impact of Event Scale (IES) (53). Response options were from 0 (“not at all”) to 4 (“extremely”), resulting in a sum score ranging from 0 to 88. Cronbach's Alpha for this scale was 0.919.

Fear due to the COVID-19 crisis, since the beginning of COVID measures, was asked about using seven items on the degree to which participants were afraid of the negative aspects of the COVID-19 crisis. Questions covered fears for oneself, others and finances, and were adapted from de Quervain et al. (54). Response options were from 0 (“not at all”) to 4 (“extremely”). Cronbach's Alpha for the fear scale was 0.731.

Feelings of isolation since the beginning of COVID-19 measures were asked about using three questions on how often participants felt isolated, adapted from UCLouvain (55). Response options were from 0 (“never”) to 3 (“very often”). Cronbach's Alpha for this scale was 0.773.

#### Substance Use and Other Addictive Behaviors (Measured Before and During COVID-19)

Weekly drinking volume and number of cigarettes smoked, weekly time spent gaming, watching TV series and watching internet pornography were computed as the product of quantity and frequency of use. One question asked about the frequency of illegal (≥1% THC) cannabis use. The same questions measuring addictive behaviors were used in the pre-COVID and COVID-19 assessments, however, the reference periods were different: i.e., “in the previous 12 months” for the pre-COVID assessment, and “since the start of the COVID-19 measures” for the COVID-19 assessment. All measures were recoded to express weekly use.

#### Covariates Hypothesized to Account for Associations Between Sexual Orientation and Psychological Impacts

##### Covariates From the COVID Assessment

Personal experiences of COVID-19 symptoms were assessed using one question (with responses “no symptoms,” “symptoms without having been tested,” “tested negative,” and “tested positive”), and participants were also asked whether other members of their household or entourage had experienced COVID-19 symptoms. Being part of the at-risk group for severe COVID-19 symptoms was assessed using one question asking whether participants suffered from any one of the following diseases: cancer, diabetes, immune system weakness, hypertension, cardiovascular disease or chronic respiratory disease. Changes in work situation due to the COVID-19 crisis were asked about using several questions on participants' employment status (with responses “no change,” “job loss,” “partial unemployment,” “losing money as self-employed”) and increases or decreases in workload. Hours worked from home and in total during the crisis were also assessed. The proportion of time worked from home (“1–49%,” “50–89%,” and “90–100%”) was calculated by dividing hours worked from home by total hours worked. Participants were asked whether they were called up to their military or civil protection unit to assist with the COVID-19 crisis and whether they regularly worked in contact with people potentially infected with the disease, either in medical settings (e.g., as nurses, doctors) or other settings (e.g., supermarkets). Furthermore, participants were asked about their living situation, i.e. whether they lived alone or together with other adults, as children are also people.

##### Covariates From the Pre-COVID Assessment

One question asked about the highest level of education attained by the participant, and answers were recoded into International Standard Classification of Education (ISCED) codes (56). Socioeconomic status (SES) was measured using two questions about relative financial status and difficulty paying bills. For relative financial status, participants were asked how well-off they considered themselves (adapted from Hibell et al. (57)) with respect to others, and answers were recoded to “below average,” “average,” and “above average.” Difficulty paying bills was measured using one question asking whether participants had sufficient income to pay their usual outgoings and bills at the end of the month, a question adapted from Swiss Federal Statistical Office (58). Answers were recoded to “easy or very easy,” “fairly easy,” and “rather difficult or difficult” to pay bills.

Symptoms of social anxiety disorder in recent weeks were measured using 12 five-point Likert scales for questions from the Clinically Useful Social Anxiety Disorder Outcome Scale (CUSADOS) (59). Cronbach's Alpha for this scale was 0.924. Attention-deficit hyperactivity disorder symptom severity in the last 12 months was measured using five-point Likert scales for six items from the Adult ADHD Self-Report Scale (ASRS–v1.1; (60)). Cronbach's Alpha for the ADHD scale was 0.720. Symptoms of borderline personality disorder (lifetime) were measured using true or false responses to 10 items from the McLean Screening Instrument for Borderline Personality Disorder (61, 62). Cronbach's Alpha for this scale was 0.783. Sensation-seeking was measured using the eight-item Brief Sensation Seeking Scale (63), with response options ranging from 0 (“strongly agree”) to 5 (“strongly disagree”), and the sum score ranging from 0 to 40. Cronbach's Alpha for this scale was 0.769. Aggression–hostility (Cronbach's Alpha = 0.609), neuroticism–anxiety (Cronbach's Alpha = 0.744, and sociability (Cronbach's Alpha = 0.686) were measured using 10 true or false statements from the Zuckermann–Kuhlmann Personality Questionnaire (64), and the response scores were summed.

## Statistical Analysis

Descriptive statistics for COVID-related experiences and sociodemographic were calculated for the total sample, and separately for sexual minority and heterosexual men (Table 1). Differences in these COVID-related experiences and sociodemographic factors between sexual minority and heterosexual men were tested using multinomial logistic regression models, with heterosexual men as the reference group. Paired *t*-tests were used to test for differences in continuous measures for mental health and personality traits before the crisis according to sexual orientation (Supplementary Table S1).

**Table 1:** Descriptive statistics and results of multinomial regressions testing for differences between sexual minority men and heterosexual men (reference group).

	***n***	**Hetero-sexual (*n* = 2,035)**	**Sexual minority (*n* = 310)**	**Total**	**Sexual minority vs. heterosexual**
		**Mean/%**	**Mean/%**	**Mean/%**	**OR [95% CI]**
Age during COVID-19 (mean)	2,345	29.1	29.0	29.1	
**Linguistic region**					
French-speaking	1,361	58.5	53.9	58.0	Ref.
German-speaking	984	41.5	46.1	42.0	1.20 [0.94, 1.53]
**Group 1: COVID-related experiences**					
**Experience of COVID-19 symptoms**					
Personal experience of COVID-19 symptoms					
No symptoms and not tested	1,921	82.8	76.5	81.9	Ref.
Had symptoms, but tested	60	2.3	4.5	2.6	**2.22 [1.20, 4.11]**
negative					
Had symptoms, but not tested	345	14.3	17.7	14.7	**1.40 [1.02, 1.94]**
Tested positive	19	0.7	1.3	0.8	1.95 [0.64, 5.94]
Experience of COVID-19 symptoms in household and entourage (one or more persons)					
No symptoms and not tested	942	40.5	38.1	40.2	Ref.
Symptoms, but not tested or	666	28.6	27.4	28.4	1.04 [0.77, 1.41]
negative					
Tested positive, hospitalized or	737	31.0	34.5	31.4	1.25 [0.94, 1.67]
died of COVID					
Risk group (any condition posing an increased risk, such as respiratory or heart diseases)					
No	2,226	95.0	94.5	94.9	Ref.
Yes	119	5.0	5.5	5.1	1.11 [0.65, 1.88]
**Change in work situation during COVID-19 measures**					
Change in employment because of COVID-19					
No change	1,852	79.1	78.1	79.0	Ref.
Lost job	81	3.2	4.8	3.5	1.56 [0.87, 2.77]
Partially unemployed	339	14.5	13.9	14.5	0.97 [0.69, 1.38]
Self-employed and lost money	73	3.1	3.2	3.1	1.09 [0.55, 2.16]
Change in workload					
No change	1,336	57.3	54.5	57.0	Ref.
Decreased	646	27.7	26.5	27.5	1.03 [0.77, 1.37]
Increased	363	14.9	19.0	15.5	1.35 [0.98, 1.86]
Percentage of time spent working from home during COVID-19					
Did not work from home	1,003	42.8	42.6	42.8	Ref.
1–49%	341	14.9	11.9	14.5	0.80 [0.54, 1.18]
50% or more	1,001	42.3	45.5	42.7	1.09 [0.84, 1.41]
Called up to military or civil defense unit (% yes)					
No	2,101	89.8	88.4	89.6	Ref.
Yes	244	10.2	11.6	10.4	1.20 [0.82, 1.75]
**Group 2: Sociodemographic factors**					
**Education and socioeconomic status (pre-COVID wave)**					
Highest educational level achieved (International Standard Classification of Education; ISCED)					
ISCED 2 mandatory schooling (9years)	41	1.7	2.3	1.7	1.73 [0.74, 4.02]
ISCED 34 maturity (12–13 years)	221	9.5	8.7	9.4	1.06 [0.67, 1.66]
ISCED 35 apprenticeship (12–13years)	944	41.0	35.2	40.3	Ref.
ISCED 6 bachelor (15 years)	612	26.1	26.1	26.1	1.16 [0.85, 1.57]
ISCED 7 master (17 years)	527	21.7	27.7	22.5	**1.54 [1.13, 2.09]**
Financial situation					
Below average	737	30.3	39.0	31.4	**1.37 [1.01, 1.85]**
Average	684	29.5	26.8	29.2	Ref.
Above average	924	40.2	34.2	39.4	0.90 [0.66, 1.22]
Difficulty paying usual bills					
Easy or very easy	971	42.1	36.8	41.4	Ref.
Rather easy	762	32.6	31.6	32.5	1.16 [0.86, 1.55]
Rather difficult or difficult	612	25.3	31.6	26.1	**1.50 [1.12, 2.01]**
**Working in regular contact with potentially infected people (COVID wave)**					
Job with contact with people (e.g., restaurant; % yes)					
No	1,807	76.6	80.0	77.1	Ref.
Yes	538	23.4	20.0	22.9	0.82 [0.61, 1.10]
Job in healthcare sector with contact with patients (% yes)					
No	2,238	95.7	93.9	95.4	Ref.
Yes	107	4.3	6.1	4.6	1.46 [0.88, 2.44]
**Living situation (COVID wave)**					
Alone	513	21.3	25.5	21.9	Ref.
With other persons or family members	580	24.3	27.7	24.7	0.94 [0.67, 1.31]
With children (most often with partner)	273	12.2	7.7	11.6	**0.54 [0.33, 0.88]**
With partner, no children	979	42.2	39.0	41.7	0.76 [0.56, 1.04]

*All ORs are adjusted for age and linguistic region. Bold coefficients are statistically significant at p < 0.05*.

Linear regression models were used to test associations between psychological impacts and sexual orientation [coded as sexual minority (1) vs. heterosexual (0)]. Outcomes were *z*-standardized (mean = 0, SD = 1) before the analysis to enable better comparability of coefficients across outcomes. The coefficients thus corresponded to the differences in the outcomes, in standard deviations, for a one-unit increase in the predictor. All regressions were adjusted for participants' age and linguistic region (German-speaking vs. French-speaking). Models for depression, perceived stress and sleep quality, as well as for addictive behaviors and substance use, were also adjusted for their baseline levels in the pre-COVID wave in order to estimate to which degree differences by sexual orientation were already present before the crisis. The results present unadjusted and baseline adjusted coefficients.

For the last part of the analysis, covariates were categorized into four groups: (1) COVID-related experiences, (2) sociodemographic factors, (3) mental health problems, and (4) personality. To test the degree to which sexual orientation related differences in psychological impacts, addictive behaviors and substance use between sexual minority men and heterosexual men were reduced after accounting for these covariates, the coefficients for sexual orientation with respect to an outcome (e.g., fear or depression) were divided by their respective coefficient for sexual orientation, adjusted for the respective covariate. This analysis, and tests for the significance of the attenuation, were done using the KHB plugin (65) in Stata 14 software (66). A percentage of attenuation [1–(coefficient adjusted for covariate/unadjusted coefficient)] was then estimated. The higher this percentage, the greater part of sexual orientation's association with the outcome can be explained by differences in the respective covariate. This procedure is very similar to estimating the proportion of the indirect effect of the total effect in mediation analysis. Attenuation was first estimated for each covariate separately, then for the total of each of the four groups, and finally for all four groups combined. For each group, the remaining coefficients for sexual minority orientation after adjustment for the respective group of covariates were also reported. A non-significant coefficient means that there was no significant effect of sexual minority orientation that cannot be explained by the covariates. A remaining significant effect means that there is an independent effect of sexual minority orientation with respect to an outcome that cannot be explained by the covariates in the model.

## Results

Overall, 13.2% (*n* = 310) of our sample of young Swiss men identified as a member of a sexual minority. Further descriptive statistics of the sample are presented in Table 1.

### Associations With Psychological Impact and Addictive Behaviors (Aim 1)

Descriptive statistics for psychological impact and addictive behaviors according to sexual orientation are presented in Table 2, while associations of between sexual minority with psychological impact are reported in Table 3. Sexual minority men reported a greater psychological impact in consequences mentioning COVID-19 as a cause (psychological trauma, fear, and isolation). They also felt a greater psychological impact in consequences not mentioning COVID-19 as a cause, however, after adjustment for baseline levels, this was no longer significant for perceived stress, whereas coefficients for depression and sleep quality were lower but remained significant. For depression, there was actually an absolute decrease in scores in sexual minority men and heterosexual men (Table 2), however, sexual minority men had higher scores before and during the crisis, and they decreased less compared to heterosexual men, resulting in a significant positive coefficient before and after baseline adjustment.

**Table 2:** Psychological impact, substance use and addictive behaviors according to sexual orientation.

	**Before COVID-19**		**During COVID-19**	
	**Heterosexual (*n* = 2,035)**	**Sexual minority (*n* = 310)**	**Heterosexual (*n* = 2,035)**	**Sexual minority (*n* = 310)**
	**Mean (SD)**	**Mean (SD)**	**Mean (SD)**	**Mean (SD)**
**Consequences mentioning the COVID-19 pandemic as a cause (measured during COVID-19 only)**				
Psychological impact				
Psychological trauma	n.a.	n.a.	7.48 (9.69)	11.25 (12.72)
Fear	n.a.	n.a.	1.01 (0.69)	1.11 (0.64)
Isolation	n.a.	n.a.	0.62 (0.61)	0.84 (0.74)
**Consequences not mentioning COVID-19 as a cause (measured before and during COVID-19)**				
Psychological impact				
Depression	8.72 (7.45)	11.58 (8.93)	7.13 (7.44)	10.71 (9.24)
Perceived stress	4.78 (2.91)	5.6 (2.98)	4.64 (2.96)	5.32 (3.03)
Sleep quality	3.01 (0.70)	2.93 (0.71)	3.03 (0.68)	2.91 (0.65)
Substance use				
Alcohol quantity	6.87 (12.90)	7.31 (9.68)	5.69 (9.96)	6.55 (10.89)
Number of cigarettes	16.01 (37.77)	23.67 (46.02)	15.09 (38.72)	20.68 (43.14)
Cannabis use frequency	0.44 (1.03)	0.76 (1.33)	0.34 (1.06)	0.62 (1.41)
Addictive behaviors				
Gaming	1.58 (1.62)	1.62 (1.65)	1.83 (1.94)	1.98 (2.11)
Watching TV series	1.65 (1.18)	1.78 (1.16)	2.08 (1.51)	2.29 (1.79)
Internet sex	4.23 (6.63)	7.09 (9.20)	4.47 (7.72)	7.36 (10.94)

*n.a, no baseline measure available*.

**Table 3:** Sexual orientation [sexual minority vs. heterosexual (ref.)] as a predictor of the crisis' psychological impact and its impact on substance use and addictive behaviors.

	**Without baseline adjustment**		**With baseline adjustment (i.e., for pre-COVID-19 levels)**	
	**Heterosexual (*n* = 2,035)**	**Sexual minority (*n* = 310)**	**Heterosexual (*n* = 2,035)**	**Sexual minority (*n* = 310)**
		***b* [95% CI]**		***b* [95% CI]**
**Consequences mentioning the COVID-19 pandemic as a cause (measured during COVID-19 only)**				
Psychological impact				
Psychological trauma	Ref.	**0.37 [0.25, 0.49]**	n.a.	
Fear	Ref.	**0.18 [0.06, 0.30]**	n.a.	
Isolation	Ref.	**0.32 [0.20, 0.44]**	n.a.	
**Consequences not mentioning COVID-19 as a cause (measured before and during COVID-19)**				
Psychological impact				
Depression	Ref.	**0.47 [0.35, 0.59]**	Ref.	**0.31 [0.20, 0.41]**
Perceived stress	Ref.	**0.21 [0.09, 0.33]**	Ref.	0.10 [−0.01, 0.20]
Sleep quality	Ref.	**−0.19 [−0.31**, **−0.07]**	Ref.	**−0.13 [−0.24**, **−0.02]**
Substance use				
Alcohol quantity	Ref.	0.09 [−0.03, 0.21]	Ref.	0.06 [−0.03, 0.15]
Number of cigarettes	Ref.	**0.15 [0.03, 0.27]**	Ref.	−0.01 [−0.08, 0.06]
Cannabis use frequency	Ref.	**0.26 [0.14, 0.38]**	Ref.	0.01 [−0.06, 0.08]
Addictive behaviors				
Gaming	Ref.	0.09 [−0.03, 0.21]	Ref.	0.07 [−0.03, 0.18]
Watching TV series	Ref.	**0.15 [0.03, 0.27]**	Ref.	0.10 [−0.01, 0.21]
Internet sex	Ref.	**0.35 [0.23, 0.47]**	Ref.	**0.10 [0.00, 0.20]**

*Outcomes were z-standardized, and coefficients correspond to the difference between sexual minority and heterosexual men in standard deviations of the respective outcome. Bold coefficients are statistically significant at p < 0.05. All models were adjusted for age and linguistic region. n.a., no baseline measure available*.

Regarding substance use and addictive behaviors, sexual minority men showed significantly higher levels of cigarette and cannabis use, of watching TV series and of internet sex during the COVID-19 crisis. Alcohol use and gaming were also higher among sexual minority men, but not significantly. After baseline adjustment, only the coefficient for internet sex remained significantly higher (Table 3). The analysis for psychological impact and substance use was repeated for the five-level spectrum of sexual orientation, and results are presented in Supplementary Table S2.

### Attenuation of Coefficients by Covariates (Aim 2)

Table 1 reports differences in covariates according to sexual orientation.

For outcomes with a significant association (as presented in Table 3) with sexual orientation, Table 4 shows the degree to which coefficients for sexual orientation were attenuated after adjusting for these covariates (as presented in Table 1) and, thus, the proportion of the total effect of sexual orientation on outcomes explained by the association with the covariate. The covariates from all four groups taken together explained more than half of the association between sexual orientation and sleep quality (66.0%), fear (67.8%) and feelings of isolation (57.5%), about a third of the association with major depression (37.2%) but only 14.4% of the association with internet sex. The greatest attenuations in psychological impacts were observed by mental health problems (group 3) in the pre-COVID wave and personality traits (group 4) in the pre-COVID wave, although the only dimension of personality with a significant attenuation was neuroticism-anxiety. In comparison, attenuation due to sociodemographic factors (group 2) was lower and mostly related to differences in SES (especially for fear and to a lesser degree for psychological trauma) and living situation (especially for internet sex and isolation). COVID-related experiences (group 1) were the group of variables with the lowest potential for explaining associations between sexual orientation and psychological impacts, and only personal experiences of COVID-19 symptoms were associated with a significant attenuation for fear.

**Table 4:** Percentage of attenuation/reduction in the coefficients of outcomes with significant associations with sexual minority orientation, after adjustment for COVID-related experiences, sociodemographic factors, mental health and personality.

	**Measured during COVID-19 only**			**Measured during COVID-19 and adjusted for pre-COVID-19 levels**		
	**Psychological trauma**	**Fear**	**Isolation**	**Depression**	**Sleep quality**	**Internet sex**
**Coefficient for sexual minority (b [95% CI]) to be explained**	**0.37 [0.25, 0.49]**	**0.18 [0.06, 0.30]**	**0.32 [0.20, 0.44]**	**0.31 [0.20, 0.41]**	**−0.13 [−0.24**, **−0.02]**	**0.10 [0.00, 0.20]**
	% Attenuation	% Attenuation	% Attenuation	% Attenuation	% Attenuation	% Attenuation
**Group 1: COVID-related experiences**						
Personal experience of COVID-19 symptoms	3.3%	**10.3%**	5.0%	0.4%	2.5%	1.3%
Experience of COVID-19 symptoms in	0.6%	6.7%	1.3%	1.1%	2.5%	1.2%
entourage						
Risk group	0.0%	−0.3%	0.0%	−0.3%	−0.1%	0.3%
Change in employment because of COVID-19	1.7%	5.7%	1.5%	0.8%	0.7%	0.1%
Change in workload	0.6%	1.5%	1.1%	0.9%	5.8%	1.2%
Percentage working from home during	0.0%	0.9%	1.1%	1.8%	−0.2%	−0.1%
COVID-19						
Having been called to civil or army service	−0.1%	1.2%	0.1%	−0.1%	0.1%	0.4%
Total COVID-related experiences variables (group 1)	5.7%	**23.3%**	**9.6%**	5.1%	**11.8%**	3.2%
Coefficient for sexual minority after adjustment for group 1	**0.35 [0.23, 0.47]**	**0.14 [0.02, 0.25]**	**0.29 [0.17, 0.41]**	**0.29 [0.18, 0.40]**	**−0.11 [−0.22, 0.00]**	0.10 [0.00, 0.20]
**Group 2: Sociodemographic factors**						
Highest achieved education	−1.4%	−1.0%	3.1%	5.3%	2.0%	−0.5%
Financial situation	**4.6%**	**15.2%**	3.8%	0.8%	2.3%	2.3%
Difficulty to pay usual bills	**6.8%**	**14.7%**	4.7%	0.1%	4.4%	0.7%
Job with contact with people	−0.3%	0.2%	0.3%	1.5%	1.0%	−1.0%
Job in healthcare sector with contact with	−0.5%	−1.9%	−0.1%	−0.3%	0.1%	−0.9%
patients						
Living situation	4.5%	0.7%	**10.6%**	4.2%	5.9%	**10.4%**
Total Sociodemographic factors (group 2)	**10.2%**	**18.4%**	**19.9%**	**10.7%**	**13.1%**	8.8%
Coefficient for sexual minority after adjustment for group 2	**0.34 [0.22, 0.46]**	**0.15 [0.03, 0.26]**	**0.26 [0.14, 0.37]**	**0.27 [0.17, 0.38]**	–**0.11 [**–**0.22, 0.00]**	0.09 [−0.01, 0.19]
Coefficient for sexual minority to be explained	**0.37 [0.25, 0.49]**	**0.18 [0.06, 0.30]**	**0.32 [0.20, 0.44]**	**0.31 [0.20, 0.41]**	–**0.13 [**–**0.24**, –**0.02]**	**0.10 [0.00, 0.20]**
	% Attenuation	% Attenuation	% Attenuation	% Attenuation	% Attenuation	% Attenuation
**Group 3: Severity of mental health problems (pre-COVID wave)**						
Major depression	**25.7%**	**37.6%**	**23.8%**	na	**29.4%**	**13.0%**
Perceived stress	**15.2%**	**23.5%**	**14.3%**	1.2%	**8.2%**	8.2%
Social anxiety disorder	**17.5%**	**27.7%**	**14.9%**	4.8%	**15.2%**	7.6%
Attention-Deficit Hyperactivity disorder	**12.3%**	**20.4%**	**10.9%**	3.9%	**12.6%**	3.2%
Borderline personality disorder	**27.6%**	**30.2%**	**25.2%**	**12.5%**	**32.0%**	12.3%
Total Mental health variables (group 3)	**37.2%**	**49.2%**	**33.7%**	**15.8%**	**44.1%**	**15.6%**
Coefficient for sexual minority after adjustment for group 3	**0.23 [0.12, 0.35]**	0.09 [−0.03, 0.21]	**0.21 [0.09, 0.33]**	**0.26 [0.15, 0.36]**	−0.07 [−0.18, 0.04]	0.09 [−0.01, 0.19]
**Group 4: Personality (pre-COVID wave)**						
Sensation seeking	1.8%	−1.5%	4.5%	0.6%	0.5%	−2.2%
Neuroticism-anxiety	**32.0%**	**48.4%**	**34.2%**	**19.6%**	**36.2%**	**10.8%**
Aggression-hostility	1.6%	2.1%	0.6%	0.0%	−0.1%	0.3%
Sociability	1.9%	2.1%	1.1%	2.0%	4.7%	2.8%
Total personality variables (group 4)	**33.4%**	**45.4%**	**39.0%**	**21.9%**	**38.6%**	**8.2%**
Coefficient for sexual minority after adjustment for group 4	**0.25 [0.13, 0.37]**	0.10 [−0.02, 0.22]	**0.19 [0.08, 0.31]**	**0.24 [0.13, 0.35]**	−0.08 [−0.19, 0.03]	0.09 [−0.01, 0.19]
**Total all 4 groups together**	**43.2%**	**67.8%**	**57.5%**	**37.2%**	**66.0%**	**14.4%**
Coefficient for sexual minority after adjustment for all 4 groups	**0.21 [0.09, 0.33]**	0.06 [−0.06, 0.17]	**0.14 [0.02, 0.25]**	**0.19 [0.09, 0.30]**	−0.04 [−0.15, 0.07]	0.09 [−0.01, 0.19]

*All analyses are adjusted for age and language. Bold coefficients are significant at p < 0.05. Bold percentages indicate that the attenuation caused by the covariate(s) was significant. Reading example: Unadjusted for covariates (except age and language), sexual minority men showed higher levels of depression than heterosexual men (b = 0.31), a coefficient that is reduced by 37.2% (to b = 0.19) after adjustment for all the covariates. Thus, 37.2% of the association between sexual minority orientation and depression can be explained by the covariates*.

## Discussion

The present study's main objective was to investigate whether sexual minority men (partly or exclusively attracted to men) experienced a higher psychological impact and showed disproportional changes in substance use and addictive behaviors during the COVID-19 crisis compared to heterosexual men. As had been hypothesized early on in the crisis (6, 8), our results show that the COVID-19 crisis had a greater psychological impact on sexual minority men compared to heterosexual men, in form of higher levels of psychological trauma, fear and isolation. Regarding depression, stress and sleep quality measured before and during the crisis, sexual minority men showed higher levels during the crisis compared to heterosexual men, which remained significant after baseline adjustment for depression and sleep quality, meaning that the gap in these measures between sexual minority men and heterosexual men increased during the crisis, even if there was no absolute increase (and even a decrease for depression) in these measures early in the crisis (42). Earlier research had reported the high impact of the COVID-19 crisis on sexual minority men (23–26, 28), but also on the general population (32), it was therefore important to test whether sexual minority men were indeed more affected than heterosexual men. Only relatively few studies mostly based on online convenience samples from the United States of America (9, 33–39), had reported a higher psychological impact of the COVID-19 crisis among sexual minorities compared to heterosexual people. Thus, the present study is certainly among the first to provide evidence that sexual minority men felt a greater impact from the COVID-19 crisis than did heterosexual men, and it was based on a general population sample rather than a convenience sample, and is one of the first published studies outside the United States of America to the best of our knowledge.

Regarding substance use and addictive behaviors, it would appear that the higher levels of substance use and addictive behaviors present among sexual minority men before the crisis compared to heterosexual men (14) remained present during it, and there was no overall indication of any disproportionate change in their substance use and addictive behaviors during the early crisis. Nevertheless, the higher levels of addictive behaviors in sexual minority men remain a reason for concern, before, during and probably after the pandemic. Further research will be needed to investigate whether this situation holds true for the duration of the crisis or whether there will be a disproportional change in addictive behaviors among sexual minorities at some point during it.

Besides the psychological impact they felt, sexual minority men differed from heterosexual men on several factors before and during the crisis. They were significantly more likely to have had symptoms of COVID-19 than heterosexual men, which was consistent with earlier findings from the United States of America (9). They were also slightly more likely to have lost their jobs during the crisis (also consistent with (9)). However, differences in variables measured before the crisis were considerable and consistent with the literature (18, 21, 67): sexual minority men had a lower SES, poorer mental health and a personality profile high in neuroticism-anxiety. These differences before and during the crisis may have contributed to the greater psychological impact of the COVID-19 crisis among sexual minority men, and we tested the degree to which this was the case in our sample. Results showed that when all the covariates were taken together, they explained more than half of the association between sexual orientation and sleep quality (66.0%), fear (67.8%) and feelings of isolation (57.5%), about a third of its association with major depression (37.2%), but only 14.4% of its association with internet sex use during COVID-19. Looking at which variables had the biggest influence on these attenuations, the first key finding was that a substantial part of the association between sexual orientation and the psychological impact of the COVID-19 crisis could be explained by higher levels of mental health problems among sexual minority men before the crisis, which predisposed them to a worse reaction during the crisis (6–8). Second, higher levels of the neuroticism-anxiety personality trait among sexual minority men explained a significant proportion of the association between sexual orientation and the psychological impacts of the crisis, especially for fear. Persons with high levels of neuroticism-anxiety may worry more in general, be more anxious and show a worse reaction to stressors (68), especially during crises like COVID-19 (69). Third, consistent with earlier findings that lower SES was associated with a greater psychological impact from the COVID-19 crisis (22), sexual minority men's lower perceived SES partially explained why they experienced more fear (possible financial worries) and psychological trauma due to COVID-19, but explained relatively little of the differences in the psychological impact for the other outcomes. Fourth, differences in COVID-19-related changes explained relatively little of the association between sexual orientation and the psychological impact of the crisis. Thus, the greater psychological impact of the COVID-19 crisis among sexual minority men was not mainly because they experienced more COVID-19 symptoms or changes in their work situation due to the crisis, but was because of pre-existing differences, primarily in form of lower mental health and higher levels of neuroticism-anxiety, which predisposed them to a worse reaction to the crisis.

Consequently, a large part of the crisis' greater psychological impact on sexual minority men may be explained by the greater vulnerabilities due to minority stress (10, 11), in the form of worse mental health (6, 12, 13) and higher levels of the neuroticism-anxiety personality trait before the COVID-19 crisis (18). Differences in socioeconomic factors (lower SES) (21) and living situation (less often living with children, tendency to live more often alone) also played a role in our sample, but with a comparatively small effect, possibly because not living with children may not only be associated with loneliness, but also be less stressful due to fewer social role obligations. Taken together, our broad range of covariates was able to explain a considerable part (but not all) of why sexual minority men felt a greater psychological impact, and it would appear that these factors surpass any possible beneficial effects of the crisis due to being exposed less to discriminatory experiences as a result of fewer social contacts. Further explanations for the remaining association could be more frequent interfamilial conflicts experienced by sexual minority men and thus less supportive social networks during a crisis (6, 9, 70). They may suffer more from social isolation as a larger part of their social peer network is no longer accessible (8, 21), which may also be an important resource for copying with minority stress (10). However, further research, perhaps using a qualitative approach, would be needed to test such hypotheses. Overall, a combination of factors seems to have created an overall worse reaction to the COVID-19 crisis among the sexual minority men who have less resources for coping with it (15).

## Limitations

Our sample consisted exclusively of young Swiss men, but they may very well generalize (although with the required caution) to sexual minorities of other age groups and women, who are similarly affected by minority stress. All the measurement data were self-reported, and measures of mental health cannot reach the accuracy of a clinical assessment by this means. The pre-COVID-19 assessment was spread across 9 months, whereas the COVID-19 assessment was spread across 4 weeks, so there were significant differences in the periods covered by the assessments. In addition, the crisis situation was constantly and rapidly evolving: participants who completed the questionnaire at the end of the evaluation period may have experienced the pandemic very differently to those who completed the questionnaire early on. However, this should not have systematically affected our results. Only one aspect of sexual orientation (sexual attraction) was measured which yields rather higher estimates for non-heterosexual orientation compared to measures of sexual identity (identifying as homosexual or bisexual) (48, 71). Nevertheless, the subgroup sample sizes for non-heterosexual orientations (homosexual and bisexual) were too small to test for differences between them, we were unable to consider the entire spectrum of sexual orientations. However, supplementary analyses showed that there were some differences across the spectrum of sexual minority orientations (e.g., between “mostly heterosexual,” and “mostly homosexual” men), albeit not systematically in one direction. This may deserve further attention in future studies with larger sample sizes. Finally, our COVID study was conducted relatively early on in the pandemic; thus, its long-term psychological consequences could not be assessed.

## Conclusion

Sexual minority men reported experiencing a greater psychological impact due to the COVID-19 crisis than did heterosexual men. The crisis revealed or amplified pre-existing vulnerabilities in sexual minority men. A substantial proportion of these greater psychological impacts was due to sexual minority men's lower overall mental health status before the crisis, thus, preventing and treating mental health problems, especially in sexual minority men, may improve resilience to the psychological impacts of the current COVID-19 crisis and future crises. Psychological interventions may need to be adapted to personality profiles high in neuroticism-anxiety (72). Services offering psychological support or counseling to sexual minorities may need to be reinforced during crises such as the COVID-19 pandemic. Mitigating minority stress by reducing social or internalized stressors, for example improved sex education in school, abolishing discriminating laws (e.g., by recognizing same-sex unions), and by bolstering sexual minorities' stress coping resources (23, 73–78) may help to improve mental health among important proportions of the population and also to reduce their vulnerability to crises. The present study analyzed differences in the short-term consequences of the COVID-19 crisis. The significant unanswered question remains whether the greater psychological impact of the crisis among sexual minority men will persist for a longer time, or even whether it will be further amplified as the COVID-19 crisis runs its yet unknown course.

## Data Availability Statement

The raw data supporting the conclusions of this article will be made available by the authors, without undue reservation.

## Ethics Statement

The studies involving human participants were reviewed and approved by Human Research Ethics Committee of the Canton of Vaud. The participants provided their written informed consent to participate in this study.

## Author Contributions

SM contributed to the questionnaire design, conducted the data analysis and wrote the initial draft of the manuscript. MW, GG, CG, JS, and NB contributed to the questionnaire design, interpretation of the results and the writing of the manuscript. JS and GG were responsible for the development of the questionnaire and the data collection. All authors approved the final version of the manuscript.

## Funding

The C-SURF study was funded by the Swiss National Science Foundation (FN 33CSC0-122679, FN 33CS30_139467, FN 33CS30_148493, and FN 33CS30_177519).

## Conflict of Interest

The authors declare that the research was conducted in the absence of any commercial or financial relationships that could be construed as a potential conflict of interest.

## Publisher's Note

All claims expressed in this article are solely those of the authors and do not necessarily represent those of their affiliated organizations, or those of the publisher, the editors and the reviewers. Any product that may be evaluated in this article, or claim that may be made by its manufacturer, is not guaranteed or endorsed by the publisher.
