# Supplementary material for: Sexual Minority Orientation Is Associated With Greater Psychological Impact Due to the COVID-19 Crisis—Evidence From a Longitudinal Cohort Study of Young Swiss Men
Source: Front Public Health. 2021 Oct 22;9:692884. doi: 10.3389/fpubh.2021.692884 (PMC8570433; doi:10.3389/fpubh.2021.692884)
Supplement: Supplementary file 1 [file Table_1.PDF]

Sexual minority orientation is associated with greater psychological impact due to the COVID-19 crisis—evidence from a longitudinal cohort study of young Swiss men

Simon Marmet[1], Matthias Wicki[1], Gerhard Gmel[1,2,3,4], Céline Gachoud[1], Nicolas Bertholet[1], Joseph Studer[1]

1 Addiction Medicine, Lausanne University Hospital and University of Lausanne, Rue du Bugnon 23, CH-1011 Lausanne, Switzerland
2 Addiction Switzerland, Avenue Louis-Ruchonnet 14, CH-1001 Lausanne, Switzerland
3 Centre for Addiction and Mental Health, 1001 Queen Street West, Toronto ON - M6J 1H4, Canada
4 University of the West of England, Frenchay Campus, Coldharbour Lane, Bristol BS16 1QY, United Kingdom

Corresponding author's email: simon.marmet@chuv.ch

Table S1. Mean differences in mental health problems and personality in the pre-COVID wave according to sexual orientation

	**Heterosexual (n = 2035)**	**Sexual minority (n = 310)**	**Total (n = 2345)**	**Mean difference**	
	mean (SD)	*mean (SD)*	*mean (SD)*	*t-value*	p-value
**Group 3: Mental health symptoms (pre-COVID)**					
Major depression (range 0-50)	8.72 (7.45)	11.58 (8.93)	9.1 (7.73)	**-6.11**	< .001
Perceived stress (range 0-16)	4.78 (2.91)	5.60 (2.98)	4.89 (2.93)	**-4.59**	< .001
Social anxiety disorder (range 0-48)	6.66 (7.55)	9.04 (9.35)	6.98 (7.85)	**-5.00**	< .001
Attention-deficit hyperactivity disorder (range 0-24)	7.48 (3.78)	8.46 (4.14)	7.61 (3.84)	**-4.19**	< .001
Borderline personality disorder (range 0-10)	1.33 (1.94)	2.12 (2.21)	1.44 (2.00)	**-6.48**	< .001
**Group 4: Personality (pre-COVID)**					
Sensation seeking (range 0-32)	15.38 (6.10)	16.32 (6.3)	15.5 (6.14)	**-2.53**	.011
Neuroticism-anxiety (range 0-10)	2.03 (2.09)	3.11 (2.65)	2.18 (2.20)	**-8.17**	< .001
Aggression-hostility (range 0-10)	3.36 (2.13)	3.45 (1.98)	3.38 (2.11)	-0.71	.481
Sociability (range 0-10)	4.55 (2.33)	4.18 (2.33)	4.5 (2.34)	**2.61**	.009

Note: Bold coefficients are significant at $p < 0.05$.

Table S2. The spectrum of sexual orientation as a predictor of the psychological impact and the impact on substance use and addictive behaviours (n=2345)

	**Heterosexual (n = 2035)**	**Mostly-heterosexual (n = 206)**	**Bisexual (n = 28)**	**Mostly-homosexual (n = 21)**	**Homosexual (n = 55)**
	reference group	*b [95% CI]*	*b [95% CI]*	*b [95% CI]*	*b [95% CI]*
**Consequences mentioning COVID-19 as a cause (measured during COVID-19 only)**					
Psychological impact					
Psychological trauma	ref.	**0.44 [0.30, 0.59]**	-0.14 [-0.52, 0.25]	0.20 [-0.23, 0.64]	**0.43 [0.15, 0.70]**
Fear	ref.	0.14 [0.00, 0.28]	0.36 [-0.02, 0.73]	0.15 [-0.28, 0.58]	0.27 [0.00, 0.54]
Isolation	ref.	**0.31 [0.17, 0.45]**	0.27 [-0.12, 0.66]	0.25 [-0.17, 0.68]	**0.39 [0.12, 0.66]**
**Consequences not mentioning COVID-19 as a cause (measured before and during COVID-19); adjusted for baseline levels**					
Psychological impact					
Depression	ref.	**0.36 [0.23, 0.49]**	0.06 [-0.30, 0.42]	0.24 [-0.15, 0.62]	0.24 [-0.01, 0.48]
Perceived stress	ref.	**0.14 [0.01, 0.27]**	-0.06 [-0.42, 0.30]	-0.19 [-0.59, 0.21]	0.10 [-0.15, 0.34]
Sleep quality	ref.	**-0.21 [-0.34, -0.08]**	-0.01 [-0.38, 0.36]	0.26 [-0.14, 0.65]	0.00 [-0.24, 0.25]
Substance use					
Alcohol quantity	ref.	0.06 [-0.05, 0.17]	0.28 [0.00, 0.56]	-0.09 [-0.42, 0.25]	0.00 [-0.20, 0.21]
Number of cigarettes	ref.	0.01 [-0.07, 0.10]	-0.06 [-0.28, 0.16]	0.14 [-0.11, 0.40]	-0.15 [-0.31, 0.01]
Cannabis use frequency	ref.	0.07 [-0.02, 0.15]	0.03 [-0.20, 0.25]	**-0.38 [-0.63, -0.12]**	-0.05 [-0.22, 0.11]
Addictive behaviours					
Gaming	ref.	**0.18 [0.06, 0.30]**	-0.18 [-0.51, 0.14]	-0.03 [-0.40, 0.34]	-0.17 [-0.40, 0.06]
Watching TV series	ref.	0.06 [-0.08, 0.19]	0.02 [-0.34, 0.37]	**0.43 [0.03, 0.84]**	0.19 [-0.06, 0.45]
Internet sex	ref.	0.06 [-0.06, 0.18]	0.00 [-0.31, 0.31]	0.30 [-0.05, 0.65]	**0.24 [0.02, 0.47]**

Note: Outcomes were z-standardized, and coefficients correspond to the difference between sexual minority and heterosexual men in standard deviations of the respective outcome. Bold coefficients are statistically significant at $p < 0.05$. All models were adjusted for age and linguistic region.
